# Dyed Hair and Swimming Pools: The Influence of Chlorinated and Nonchlorinated Agitated Water on Surface-Enhanced Raman Spectroscopic Analysis of Artificial Dyes on Hair

**DOI:** 10.1021/acsomega.4c06734

**Published:** 2024-10-31

**Authors:** Aidan P. Holman, Roa Elsaigh, Ragd Elsaigh, Axell Rodriguez, Dmitry Kurouski

**Affiliations:** † Interdisciplinary Faculty of Toxicology Program, Texas A&M University, College Station, Texas 77843, United States; ‡ Department of Biochemistry and Biophysics, Texas A&M University, College Station, Texas 77843, United States; § Nanomedicine Program, College of Science, Northeastern University, Boston, Massachusetts 02115, United States; ∥ Department of Biomedical Engineering, Texas A&M University, College Station, Texas 77843, United States

## Abstract

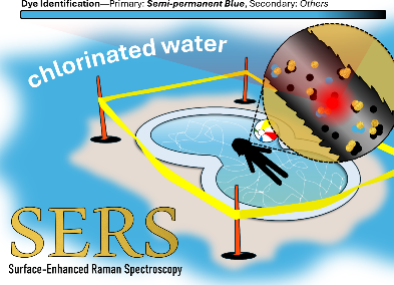

Chlorine, commonly found in pools and tap water, presents an intriguing concern in forensic hair analysis due to its sources and composition. Current forensic analysis involves optical microscopy which is subjected to advanced training where even multiple experts can deliver opposing conclusions for the same hair sample. Despite challenges in traditional analysis methods, emerging techniques like surface-enhanced Raman spectroscopy (SERS) offer promising solutions, showcasing success even in harsh environments like prolonged sunlight or stagnant water immersion. This study employs partial least-squares discriminant analysis (PLS-DA) to evaluate SERS efficacy in identifying dyes on hair immersed in chlorinated and distilled moving water for up to eight weeks. Our results demonstrated that one semipermanent colorant overwhelmingly influenced Raman signals in dyed hair exposed to both chlorinated and nonchlorinated water over an eight-week period, masking other colorants’ spectral signatures. Despite one colorant’s dominance, PLS-DA identified underlying colorants and their exposure conditions, suggesting persistent, unique interactions between original colorants and the environment. This study demonstrates the high potential for PLS-DA-based identifications of dyes on hair using SERS.

## 1 Introduction

The vibrant hues and striking transformations that hair dye can offer have long captivated the imagination of individuals seeking to express their unique personalities and styles. However, the longevity and stability of these colorful manifestations are often threatened by various environmental factors, chief among them being chlorine water.^1^ Chlorine, a widely utilized disinfectant commonly found in swimming pools and tap water supplies and thus constantly interacts with hair. The origin of free chlorine in swimming pools can be traced back to one of two primary sources. First, substantial tablets, designed for passive diffusion, are employed through specialized chlorine dispensers that can either float within the pool or connect to the water source. Second, an aqueous solution may be administered instead along the pool’s perimeter and at its deepest points. Both sources share a fundamental component, with trichloroisocyanuric acid constituting over 90% of their composition. Notably, this compound surpasses the previously favored choice, sodium hypochlorite, by offering reduced health risks and the remarkable ability to extend the presence of free chlorine.^2^ What’s more is that with the extension of free chlorine in swimming pools, the process of bleaching is also enhanced. As summer brings a surge in recreational swimming pool activities accompanied by a growing trend of people dyeing their hair, it raises the question of how chlorine-induced bleaching impacts the field of forensic hair analysis, specifically the traceability of dyes in hair.

Besides DNA analysis of hair, forensic hair analysis mainly employs forensic microscopy to reveal key information of collected hair. Determining if hair was dyed and what color can allow for the identification, matching, and narrowing down of victims and suspects in a criminal case. A widely recognized procedural manual asserts that for a definitive assessment of dyeing or bleaching, the hair’s base (bulb) must be present on the hair follicle for a comparative evaluation of color in both regions.^3^ However, this method not only suffers from subjectivity but also encounters practical challenges. The base of a hair follicle is seldom encountered at crime scenes due to its degradation over time, and it may not always remain attached to its corresponding hair follicle when separated from the scalp.^3^ Moreover, forensic microscopy of hair.

To address these limitations, an emerging analytical technique shows significant promise: surface-enhanced Raman spectroscopy (SERS) (also referred to as surface-enhanced Raman scattering spectroscopy). SERS harnesses the electromagnetic properties of colloidal nanoparticles to amplify the intensity of scattered light gathered in conventional Raman spectroscopy by a factor of around a millionfold. In 2015, Kurouski and Van Duyne showed that SERS could generate vibrant spectra from multiple dyed hair samples.^4^ Since then, research like that of studying the effects of bleach, acidulated foods and drinks, and body fluid contamination on dyed hair have built a repertoire of successes for SERS in dyed hair analysis.^5,6^ Of relevance to this research, SERS can be used to identify artificial dyes on hair in other harsh environments such as up to ten weeks in direct sunlight and twelve weeks submerged in stagnant lake water.^7,8^

In this paper, we utilize partial least-squares discriminant analysis (PLS-DA) as a tool to assess the effectiveness of SERS in accurately detecting artificial dyes on hair subjected to continuous submersion in chlorinated moving water for a duration of up to eight weeks. To distinguish the individual impacts of chlorine exposure and water agitation on dye stability, we also investigate the effects of immersing dyed hair in distilled moving water for the same duration, employing SERS. Additionally, we delve into the capabilities of SERS in uncovering distinct patterns of dye degradation within the acquired spectra. This information may prove invaluable in constructing a chronological sequence of events based on hair samples collected from both victims and suspects.

## 2 Materials and Methods

### 2.1 Hair Preparation

We used hair samples from the same source: undyed, unprocessed Caucasian female hair donated by a 21-year-old colleague who was fully informed about the use of her hair for this research. The hair was dyed with Ion brand products, specifically Ion Jet Black (permanent black), Ion Sapphire (permanent blue), Ion Blackest Black (semipermanent black), and Ion Sapphire (semipermanent blue). For the purposes of this experiment, we designated permanent black and blue as “PBA” and “PBU,” and semipermanent black and blue as “SBA” and “SBU,” respectively. The components of the hair dyes are listed in Table 1.

**Table 1:** Hair Dyes and Their Colorants and Couplers, Where Applicable

hair dye item (SBS no.)	group	colorant(s) (and *couplers*) in dyes
ion jet black (305430)	PBA	(1) 2,4-diaminophenoxyethanol (*coupler*), (2) toluene-2,5-diamine, and (3) 1-hydroxyethyl-4,5-diamino pyrazole
ion sapphire (405601)	PBU	(1) 5-amino-6-chloror-o-cresol (*coupler*) and (2) *n*,*n*-bis(2-hydroxyethyl)-*p*-phenylenediamine
ion blackest black (405079)	SBA	(1) basic blue 99, (2) basic brown 16, (3) HC blue no. 2, and (4) HC yellow no. 4
ion sapphire (405068)	SBU	(1) basic yellow 87, (2) basic blue 124, and (3) HC blue no. 15

A clean beaker was used to combine the permanent dye and activator, while a clean graduated cylinder was used to apply equal amounts of each colorant to the hair samples. We mixed the permanent dyes with Ion Sensitive Scalp Creme Developer, adhering to the manufacturer’s recommended volumes. The dye was carefully worked into the hair until each strand was fully saturated. Following the time recommended on the dye packaging, the hair was washed under low-pressure deionized water using a small stainless-steel strainer until the runoff was clear. Subsequently, the hair was allowed to air-dry. The hair for each of the two environments studied was prepared separately.

### 2.2 Chlorinated Water Procedure

A commercial chlorine pool tablet (The Clorox Company, Oakland, CA) was used to simulate pool-chlorine conditions. The tablet consisted of 94.05% trichloroisocyanuric acid (dominating ingredient) which allowed the availability of approximately 84.65% free chlorine (of the dominating ingredient) once dissolved in water. According to package instructions, one 170 g tablet should be used for every 5000 gallons of water. To comply with this, we added 2.7 mg of our chlorine tablet to 300 mL of distilled water. We used distilled instead of deionized water because metal ions have proven somewhat effective as bactericides and are not commonly filtered in pools.^9^ Stirring was set to 380 rpm (rpm) (setting “6” on a Corning PC-220 Stirring/Hot Plate) using a 2” magnetic stir bar so hair was exposed to a constant physical force against the water as experienced in actual swimming pools. Before the hair groups were added, stirring occurred to fully distribute the pool tablet solute and did not cease until the solution was homogeneous. Evaporated water was counteracted by refilling the beaker with distilled water daily to return it to the ∼300 mL final volume. All repreparations follow these same steps unless otherwise stated.

### 2.3 Nonchlorinated Water Procedure

300 mL of distilled water was added to the beaker alone prior to hair submergence. Stirring was restricted to 380 rpm using a 2” magnetic stir bar. Evaporated water was counteracted by refilling the beaker with distilled water daily to return it to the ∼300 mL final volume. All repreparations follow these same steps unless otherwise stated.

### 2.4 Sample Collection

Prepared hair samples were submerged in respective environments (chlorinated and nonchlorinated moving water) for a full week before each collection. During collection, hair groups were removed all-at-once from their water environment to snip an inch from a few hair strands that are then sealed in a plastic bag and stored in a dark environment to be later used for scanning/analysis. Removed hair groups were then resubmerged in newly preprepared water environments to comply with pool tablet package instructions (which state to add a new tablet every week). Briefly, before the hair was resubmerged during collection, the hair was gently rinsed with deionized water to remove any compounds generated by the previous water environment. Collection stopped after eight weeks of submergence.

### 2.5 Nanoparticle Preparation

Gold nanorods (AuNRs) were synthesized using published methods by Burrows et al. (2017).^10^ First, a seed solution was prepared by diluting 250 μL of 0.01 M HAuCl_4_ in 9.75 mL of 0.1 M CTAB and stirred. Then, a fresh cold solution of 0.01 M NaBH_4_ is prepared by first diluting 0.1 M NaBH_4_ in 10 mL H_2_O. Only milli-Q ultrapure H_2_O was used throughout the synthesis and collection. This will result in a honey-colored solution and left to age for at least an hour. Next, 500 μL 0.01 M HauCl_4_ is added to 9.5 mL of 0.1 M CTAB solution, followed by 20 μL 0.01 M AgNO_3_, 55 μL 0.01 M ascorbic acid, and 12 μL of the prepared seed solution. Gently stir for an hour and immediately collect to centrifuge at 4000 rpm for 30 min, or 11,000 rcf for 15 min. Discard the supernatant, and resuspend in H_2_O, repeat this process two times for a total of three washes. The AuNRs were characterized using scanning electron microscopy, Figure S1.

Chemicals utilized for synthesis: cetyltrimethylammonium bromide (CTAB, VWR International), Gold (III) chloride hydrate (HauCl_4_ x H_2_O, Aldrich), ascorbic acid (Sigma-Aldrich), sodium borohydride (NaBH_4_, Sigma-Aldrich), and silver nitrate (AgNO_3_, Sigma).

### 2.6 Raman Spectroscopy

The excitation wavelength laser light, equipment, and power were chosen based on published methods from Esparza and co-workers.^5^ SERS spectra were collected using a TE-2000U Nikon inverted confocal microscope, equipped with a 20x objective. A solid-state laser generated 785 nm light, while power through each sample was kept at 1.8 mW. Considering an average diameter of the beam on the sample surface to be around 100 μm, hair was exposed to ∼45.8 W/cm^2^ of 785 nm light. Scattered light was collected using the same magnification and directed using a 50/50 beam splitter into an IsoPlane-320 spectrometer (Princeton Instruments) equipped with a 600 groove/mm grating. Prior to entering the spectrometer, elastically scattered photons were blocked by a long-pass filter (Semrock, LP03-785RS-25). Inelastically scattered photons were collected using PIX-400BR CCD (Princeton Instruments).

Fifty spectra from each sample, comprising 15–20 spectra from each of three separate locations on a hair strand per sample group, (3,240 spectra total) were collected by placing each hair on a glass coverslip and applying ∼5 μL of the AuNR solution. The hair strand was coated with the AuNR solution by maneuvering it across the slide, ensuring the nanorod solution covered approximately 10 mm of the strand, regardless of the hair’s actual length. The laser light was strategically positioned lateral to the medulla and proximal to the point of attachment of the hair, locations that consistently yielded the most intense peaks for the bands of interest. The total acquisition times for the measurements varied between 3 and 15 s.

### 2.7 Data Analysis

PLS-DA is a widely favored chemometric technique for spectral data, particularly when compared to other methods like support vector machine (SVM), soft independent modeling of class analogy (SIMCA), and principal component analysis (PCA). PLS-DA excels in managing complex data sets that feature high multicollinearity and noise.^11^ This method integrates regression and classification by leveraging the correlation between spectral data and sample classes. Unlike binary classifiers such as SVM and SIMCA, PLS-DA is capable of handling multiclass classification, making it more suitable for complex classification tasks. Furthermore, unlike PCA, which only identifies the most significant components in the data, PLS-DA extracts latent variables that optimize the correlation between spectral datapoints and class variables, thus enhancing its performance in data sets with intricate class structures and small sample sizes.

All spectra were baseline-corrected (6th order) and area-normalized (as displayed) with their respective colorant groups (e.g., all PBA-dyed hair spectra were area-normalized separate from other colorants and recombined after normalization when necessary) before analysis using MATLAB. Chemometric analysis of acquired spectra was done in MATLAB equipped with PLS_Toolbox 9.0 (eigenvector Research, Inc., Manson, WA). For PLS-DA, cross-validations from full calibration models were employed (i.e., all spectra were used to train and test the model) unless otherwise specified. The classes and samples are equally distributed in all training and testing sets. In light of concerns surrounding model fitness for cross-validation models in PLS-DA, signal-to-noise ratio (SNR) and 1,000 permutations were evaluated, Figures S2–S4 and Tables S1–S3. Preprocessing of each PLS-DA model was done using first-derivative smoothing (*n* = 2, fl = 15 pt.) and mean centering. Latent variables (LVs) were selected based on the most appropriate root-mean-square error (in cross-validation) value for each model. Accuracy, herein, will be determined by the average of the combined true positive rate (TPR or sensitivity) and true negative rate (TNR or specificity).

## 3 Results and Discussion

### 3.1 SERS-Based Detection of Colorants

**Figure 1:**
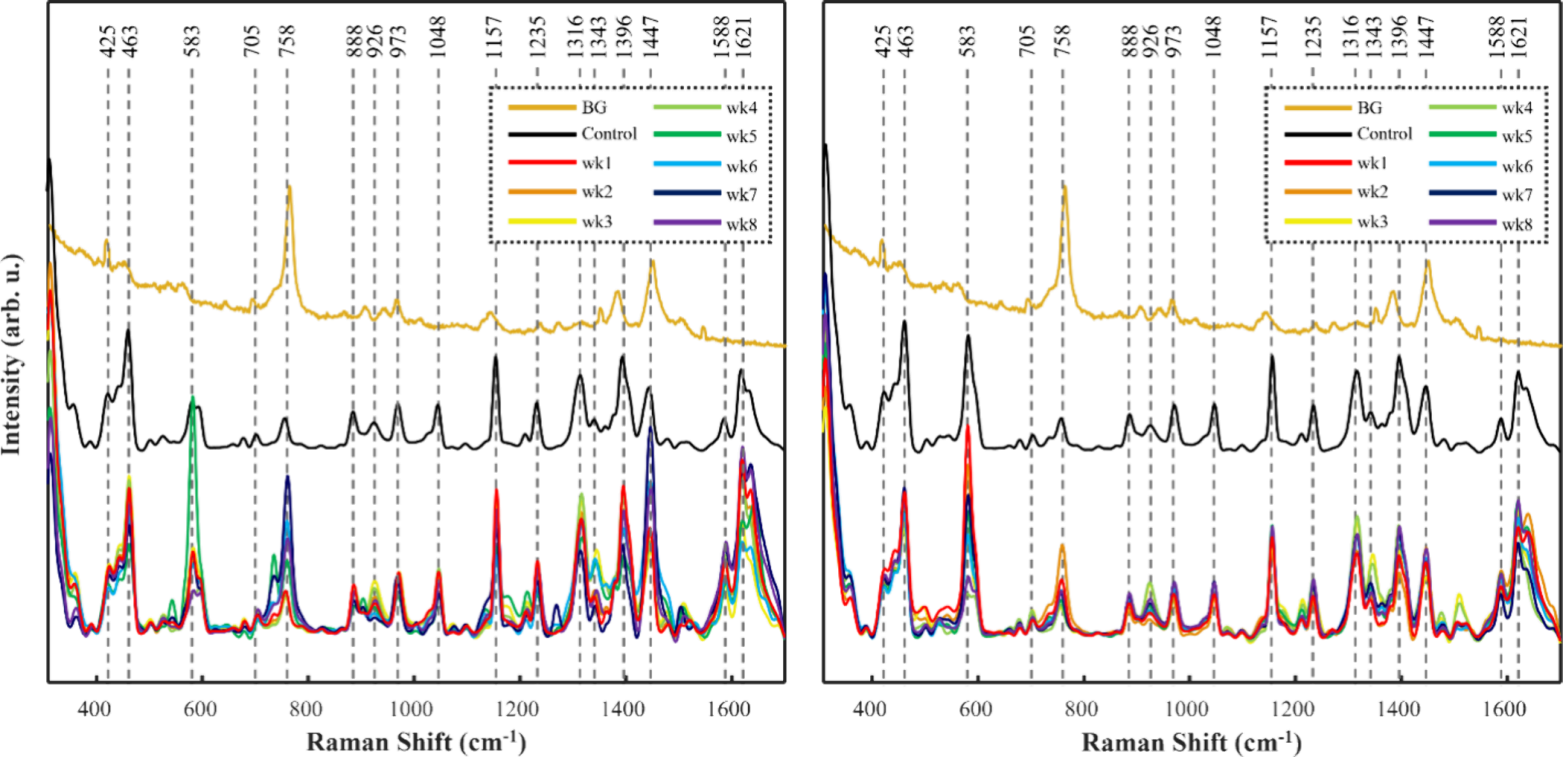
Averaged SERS spectra of SBU-dyed hair within a chlorine water environment (left) and nonchlorinated water environment (right). BG = raw, unprocessed SER spectrum of AuNRs prepared on glass coverslip; Control = SER spectra of preconditioned SBU-dyed hair.

**Figure 2:**
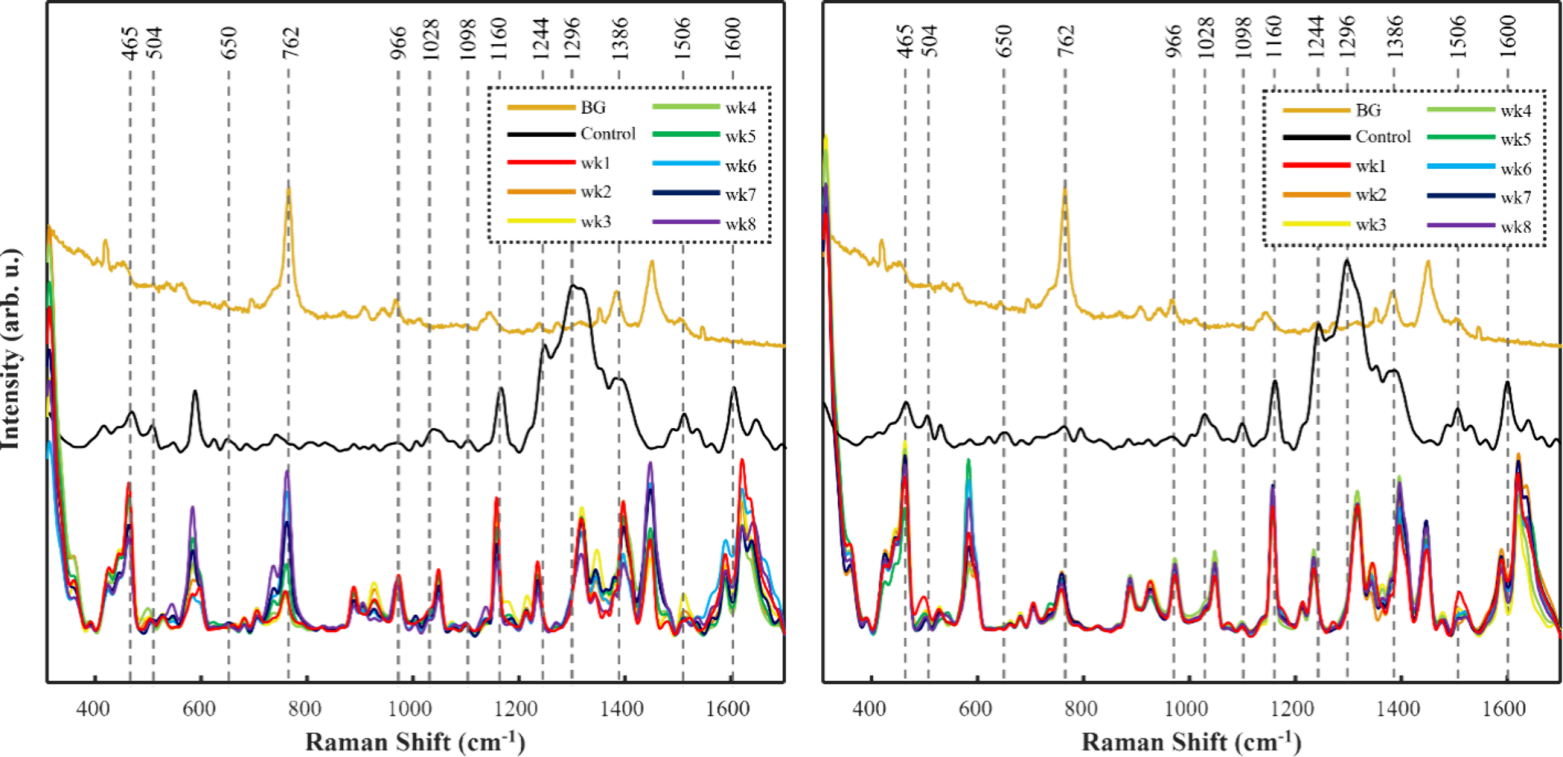
Averaged SERS spectra of SBA-dyed hair within a chlorine water environment (left) and nonchlorinated water environment (right). BG = raw, unprocessed SER spectrum of AuNRs prepared on glass coverslip; Control = SER spectra of preconditioned SBA-dyed hair.

**Figure 3:**
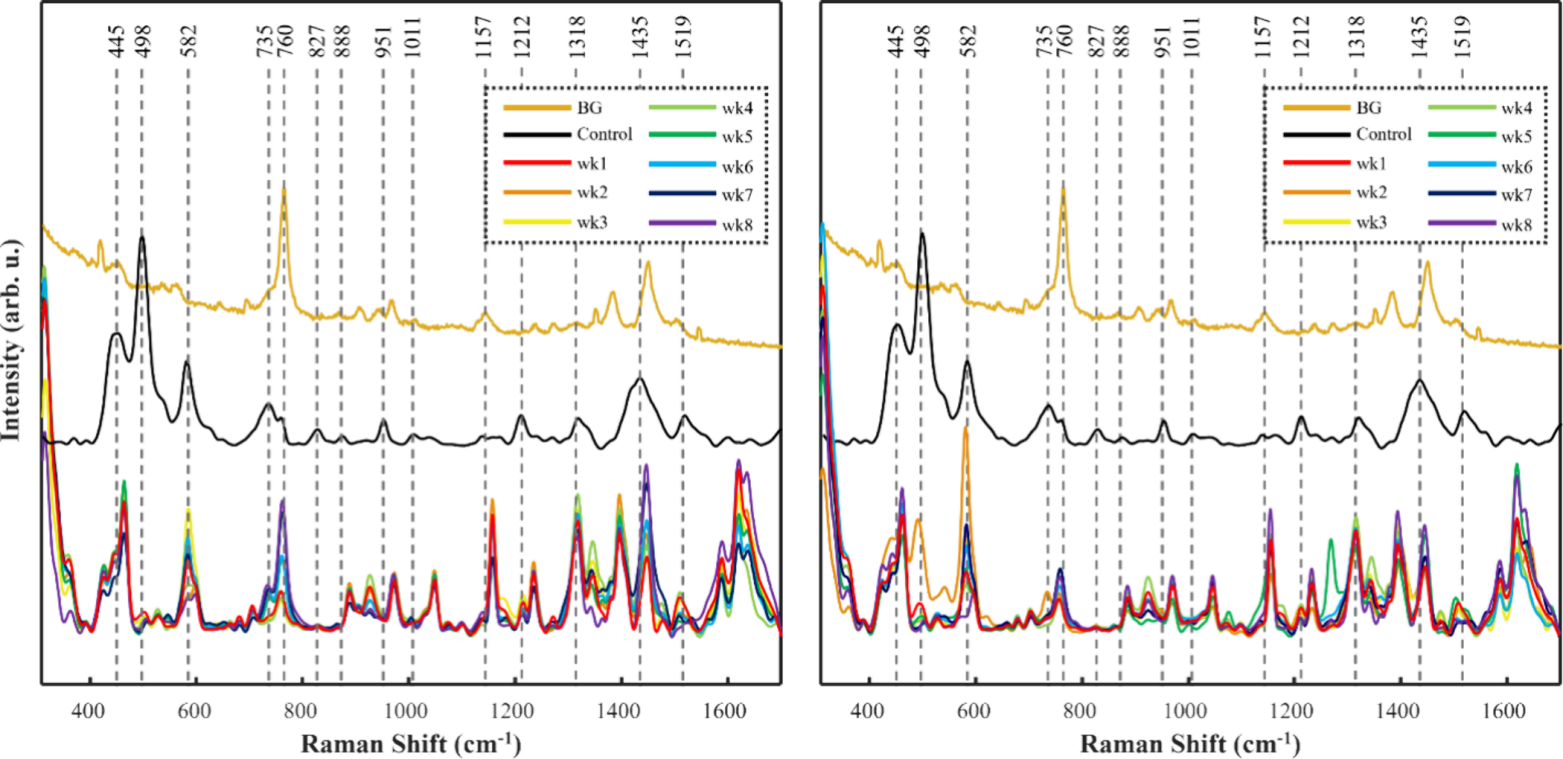
Averaged SERS spectra of PBA-dyed hair within a chlorine water environment (left) and nonchlorinated water environment (right). BG = raw, unprocessed SER spectrum of AuNRs prepared on glass coverslip; Control = SER spectra of preconditioned PBA-dyed hair.

**Figure 4:**
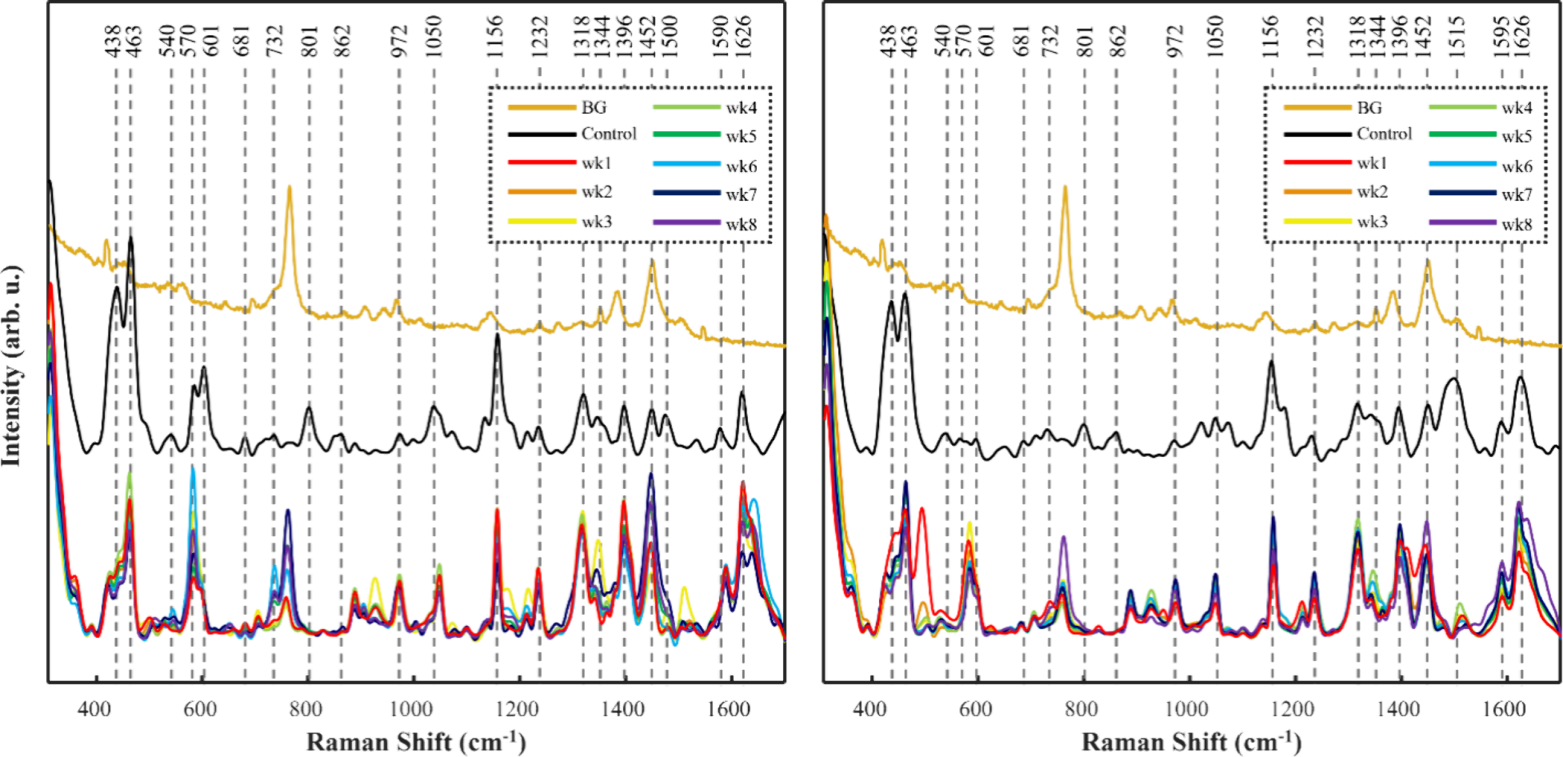
Averaged SERS spectra of PBU-dyed hair within a chlorine water environment (left) and nonchlorinated water environment (right). BG = raw, unprocessed SER spectrum of AuNRs prepared on glass coverslip; Control = SER spectra of preconditioned PBU-dyed hair.

It quickly became evident that one of the four colorants (SBU) dominated the Raman signals across all dyed hair samples starting after one week of exposure to agitated chlorinated and nonchlorinated water, Figures 1–4. We hypothesize that after the freshly dyed, dried hair was exposed to high agitation, the semipermanent colorant weakened and some of it released from the surface of the hair cuticle. Once released, it appeared to have bonded to the cuticles of all the other hairs in the same environment, masking the other colorant signatures, regardless of permanence. Indeed, all vibrational modes for SBU-dyed hair were found in all exposed hair samples once analyzed. Previous literature has shown that the specific dye, SBU, can almost completely mask other colorant signatures whether beneath or covering the other colorant.^12^ This dominance of SBU could be also explained from a perspective of a resonance Raman effect, which is observed if the absorbance of the sample overlays with excitation wavelength used to acquire Raman spectra. Specifically, SBU absorbs in the red part of the electromagnetic spectrum, while all spectra were acquired using 785 nm excitation. Thus, Raman scattering from SBU, under our experimental conditions, would be around a million-fold stronger compared to the scattering from dyes that do not have absorption in the red part of electromagnetic spectrum. What has not been realized is the extent of the properties of the colorant to withstand weeks of chlorination and agitation, as shown herein.

To confirm the dominance of SBU after exposures, we utilized PLS-DA to classify each colorant from the combined spectra after each week’s exposure built (calibrated) with SER spectra of the preconditioned dyed hairs, Table 2. We contributed the percentage of all SER spectra mis-identified as SBU-dyed hair to the false positive rate for SBU and the number of SER spectra correctly predicted as its colorant group the true positive rate for that colorant. We found that the lowest false positive rates, 94 and 91.7%, were among week 1 and week 2 SER spectra. However, because week 1 only had a true positive rate above 0 for SBU, it is understood that any other colorant spectra that did not contribute to the SBU FPR were misidentified as other colorants. Overall, these results indicate that SBU has indeed compromised almost all of the signal of dyed hair exposed to chlorinated and nonchlorinated agitated water over prolonged times, regardless of whether it was dyed with SBU before exposure.

**Table 2:** Combined PLS-DA Validation (Test) Models’ (Calibrated with All Control Spectra) Results to Classify Dyed Hair from Both Environments (Chlorinated and Nonchlorinated Water) to Its Respective Hair Dye Group after Each Week of Exposure^a^

LVs = 9		TPR (%)			
training group	SBU FPR (%)	PBA (n = 100)	PBU (n = 100)	SBA (n = 100)	SBU (n = 100)
control	0	100	100	100	100

test groups	SBU FPR (%)	PBA (*n* = 100)	PBU (*n* = 100)	SBA (*n* = 100)	SBU (*n* = 100)
week 1	94	0	0	0	100
week 2	91.7	25	0	0	100
week 3	100	0	0	0	100
week 4	100	0	0	0	100
week 5	100	0	0	0	100
week 6	100	0	0	0	100
week 7	100	0	0	0	100
week 8	100	0	0	0	100

a LVs – latent variables; TPR – true positive rate; FPR – false positive rate; *n* – number of spectra involved per cell.

### 3.2 Are the Environmental Effects Unique?

A question that still has not been answered is whether the effects of chlorinated and nonchlorinated water are differentiable when analyzing dyed hair with SERS. Here, we accomplished this by using PLS-DA to identify hair groups that either came from chlorine water or nonchlorinated water, Table 3. We found that each model had over 99% accuracy at determining whether the dyed hair was exposed to chlorine water or not. These results show that the effects of water agitation are differentiable with the presence of bleaching agents such as chlorine.

**Table 3:** Combined PLS-DA Cross-Validation Models’ Accuracies on Identifying the Specific Colorant Group between Both Environments

group (LVs)	model accuracy (%)	predicted as chlorine-exposed; accuracy (%)	predicted as nonchlorine exposed; accuracy (%)
week 1 (8)	99.8	99.8	99.8
week 2 (8)	100	100	100
week 3 (8)	99.2	99.2	99.2
week 4 (7)	100	100	100
week 5 (8)	100	100	100
week 6 (5)	99.8	99.8	99.8
week 7 (6)	100	100	100
week 8 (7)	100	100	100

While the majority of exposed dyed hair can be classified or misclassified as SBU-dyed hair, it is worth exploring if the effects of each environment can be seen in the SER spectra after prolonged exposure. To determine this, we used PLS-DA to identify the colorants between each environment by week of exposure, Table 4. We found that each model had over 95% accuracy at determining which colorant was the original group and whether that colorant was exposed to chlorinated or nonchlorinated water. These results suggest that although the SBU colorant remained on all hairs, the chemical properties associated with the underlying colorant and specific environment it was exposed to led to unique interactions. These interactions were significant enough to allow for well-differentiation throughout all weeks of exposure, suggesting that the colorant may still be detectable beyond the observed spectrum. It is worth noting, then, that a common technique used in forensic spectral signature identifications such as the characteristic peaks method, would not be plausible here.

**Table 4:** Combined PLS-DA Cross-Validation Models’ Accuracies on Identifying the Specific Colorant Group between Both Environments^a^

group (LVs)	model accuracy (%)	predicted as chlorine; accuracy (%)				predicted as other; accuracy (%)			
		PBA (*n* = 50)	PBU (*n* = 50)	SBA (*n* = 50)	SBU (*n* = 50)	PBA (*n* = 50)	PBU (*n* = 50)	SBA (*n* = 50)	SBU (*n* = 50)
week 1 (9)	99.9	99	100	100	100	100	100	99.9	100
week 2 (9)	98.2	98.2	100	87	100	100	100	100	100
week 3 (10)	95.4	91.7	98.2	94.2	87.6	100	99.6	96.8	95.4
week 4 (9)	98.7	100	100	94	100	100	99.6	96.2	100
week 5 (10)	99.4	99.9	99	100	100	100	97	99.6	100
week 6 (10)	99.4	99.9	99	100	100	100	97	99.6	100
week 7 (8)	99.4	99.6	100	96.6	100	99	100	100	100
week 8 (9)	99.3	100	100	95.9	100	100	99.4	100	99

a *n* – Number of spectra involved per cell.

### 3.3 Related Swimming Pool Exposure

To compare the assessed chlorine effects on hair under timed submergence to the actual amount of time hair is submerged by real people, we used the U.S. Environmental Protection Agency (EPA) Exposure Factors Handbook coupled with the U.S. Census Bureau Report Number Statistical Abstract, Tables 5 and 6.^13,14^

**Table 5:** Predicted Amount of Yearly Exposure to Swimming Pools by People in the United States

consumer	swimming pool activity (min/event)^13^	swimming pool activity rate (events/year)^14^	exposure frequency (min/year)	yearly exposure duration (weeks)
adult men (D50)	68	6	408	0.04
adult men (D95)	180	6	1080	0.11
adult women (D50)	67	6	402	0.04
adult women (D95)	170	6	1020	0.10
children (D50)	81	6	486	0.05
children (D95)	200	6	1200	0.12

**Table 6:** Predicted Amount of Lifetime Exposure to Swimming Pools by People in the United States^a^

consumer	child exposure duration (weeks)^b^	adult exposure duration (weeks)^b^	lifetime exposure duration (weeks)^b^
men (D50)	1.01	1.98	2.99
men (D95)	2.50	5.25	7.75
women (D50)	1.01	1.95	2.96
women (D95)	2.50	4.96	7.46

a Values displayed in the Table 4 were derived from Table 3’s “Exposure frequency.”

b Values for age were followed by EPA considerations of children to be from 0 to 21 years of age, adults to be >21 years of age, and a lifetime to be 70 years.

According to our results, SERS can be used to detect dyes on hair after a lifetime of activity in chlorine-water, albeit with low sensitivity when exposed to waters contaminated with other “aggressive” colorants that may dominate the SER spectra. This scenario, however, assumes that the person had their scalp hair dyed as soon as they were born, never bleached or redyed the hair, and all other processes done to hair such as washing, weathering, etc., did not take place or have no effect on the stability of the colorant. So, while the calculated lifetime exposure is unrealistic, in reality the average person would get their hair redyed every 4–6 weeks.^15^ So even if they spent their entire swimming activity in a year (0.04–0.12 weeks) after dying their hair again, SERS could still be used to detect colorants from chlorine-water exposed hair.

## 4 Conclusions

The experiment revealed that the colorant SBU significantly dominated the Raman signals of dyed hair exposed to agitated chlorinated and nonchlorinated water over eight weeks. This dominance was attributed to the release and subsequent bonding of SBU to the hair cuticles, effectively masking the spectral signatures of other colorants present. Despite the prolonged exposure and the compromised signals, the study demonstrated the feasibility of using PLS-DA to differentiate between dyed hair exposed to chlorinated and nonchlorinated water, with high accuracy. If one is to identify underlying colorants from hair exposed in these ways, they would not be able to use other methods such as the subjective characteristic peaks method whereby the number of peaks is compared to the known number for identifications. Instead, PLS-DA, a machine-learning model, should take the forefront for these identifications as shown. Additionally, PLS-DA allowed for the identification of underlying colorants and their exposure conditions, suggesting that unique interactions between the original colorant and the environment persisted despite the dominance of SBU. The study also highlighted the potential application of SERS in detecting dyes on hair even after prolonged exposure to chlorine water, although sensitivity may be reduced in the presence of other dominant colorants. Future research should look at using larger water bodies for similar experimentation, as ours was 100,000 times smaller than an average pool (holding 30,000 L of water). Additional research could explore using more hair dyes and different concentrations of chlorine tablets. Finally, it is important to investigate the contribution of biotic and abiotic factors, such as body fluid contaminants, temperature and UV radiation upon the direct utilization of RS in the field.^6−8^

## Supporting Information Available

Electron micrograph of AuNRs, estimated SNR for the PLS-DA models used, and result of permutation tests used in all PLS-DA models (PDF)
