# Supplementary material for: Dyed Hair and Swimming Pools: The Influence of Chlorinated and Nonchlorinated Agitated Water on Surface-Enhanced Raman Spectroscopic Analysis of Artificial Dyes on Hair
Source: ACS Omega. 2024 Oct 31;9(45):45263–9. doi: 10.1021/acsomega.4c06734 (PMC11561636; doi:10.1021/acsomega.4c06734)
Supplement: Supplementary file 1 — ao4c06734_si_001.pdf [file ao4c06734_si_001.pdf]

Dyed Hair and Swimming Pools: The Influence of Chlorinated and Non-chlorinated Agitated Water on Surface-Enhanced Raman Spectroscopic Analysis of Artificial Dyes on Hair

Aidan Holman,[1,2] Roa Elsaigh,[2] Ragd Elsaigh,[2] Axell Rodriguez,[2,3] and Dmitry Kurouski[2,4*]

*E-mail: dkurouski@tamu.edu Tel: 979-458-3778.

ORCID

Aidan Holman: 0000-0003-4244-7348

Dmitry Kurouski: 0000-0002-6040-4213

Axell Rodriguez: 0009-0006-0640-9714

1. Forensic and Investigative Sciences Program, Texas A&M University, College Station, Texas 77843, United States
2. Department of Biochemistry and Biophysics, Texas A&M University, College Station, Texas 77843, United States
3. Nanomedicine College of Science Program, Northeastern University, Boston, Massachusetts 02115, United States
4. Department of Biomedical Engineering, Texas A&M University, College Station, Texas, 77843, United States

Supporting Information

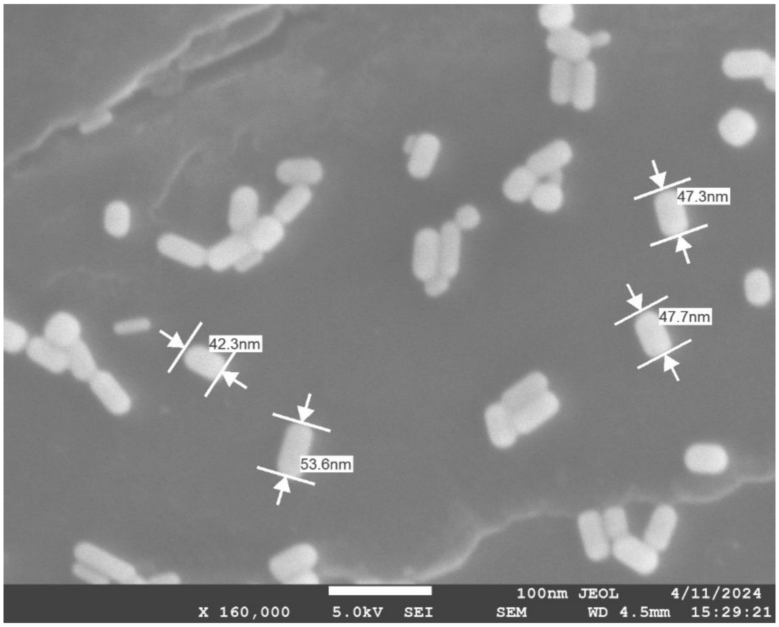

Figure S1. Scanning electron micrograph of our AuNRs.

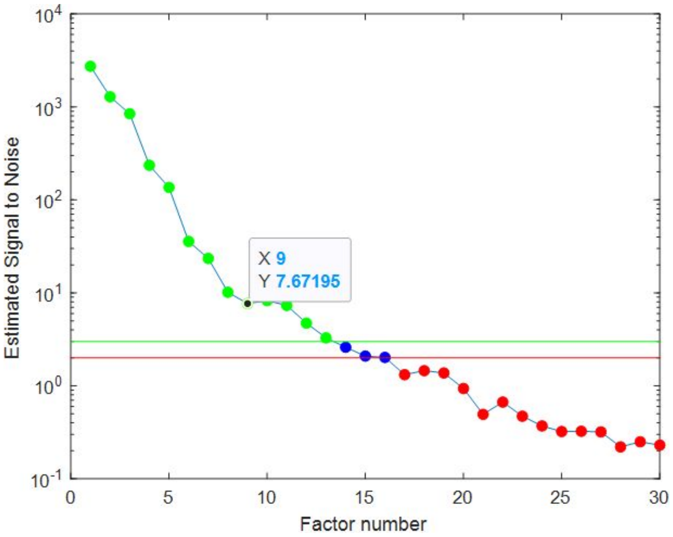

Figure S2. Estimated SNR for the PLS-DA model used in Table 3.

Table S1. Permutation test results for the PLS-DA model in Table 3, built by control spectra.

		**Random Prediction Probability (p)***			
**PLS-DA Model**	**Validation Type**	**PBA**	**PBU**	**SBA**	**SBU**
Control	Self-prediction	0.005	0.005	0.005	0.005

	Cross-validated	0.005	0.005	0.005	0.005

**If $p < 0.05$, the predictive classification of the colorant is non-random.*

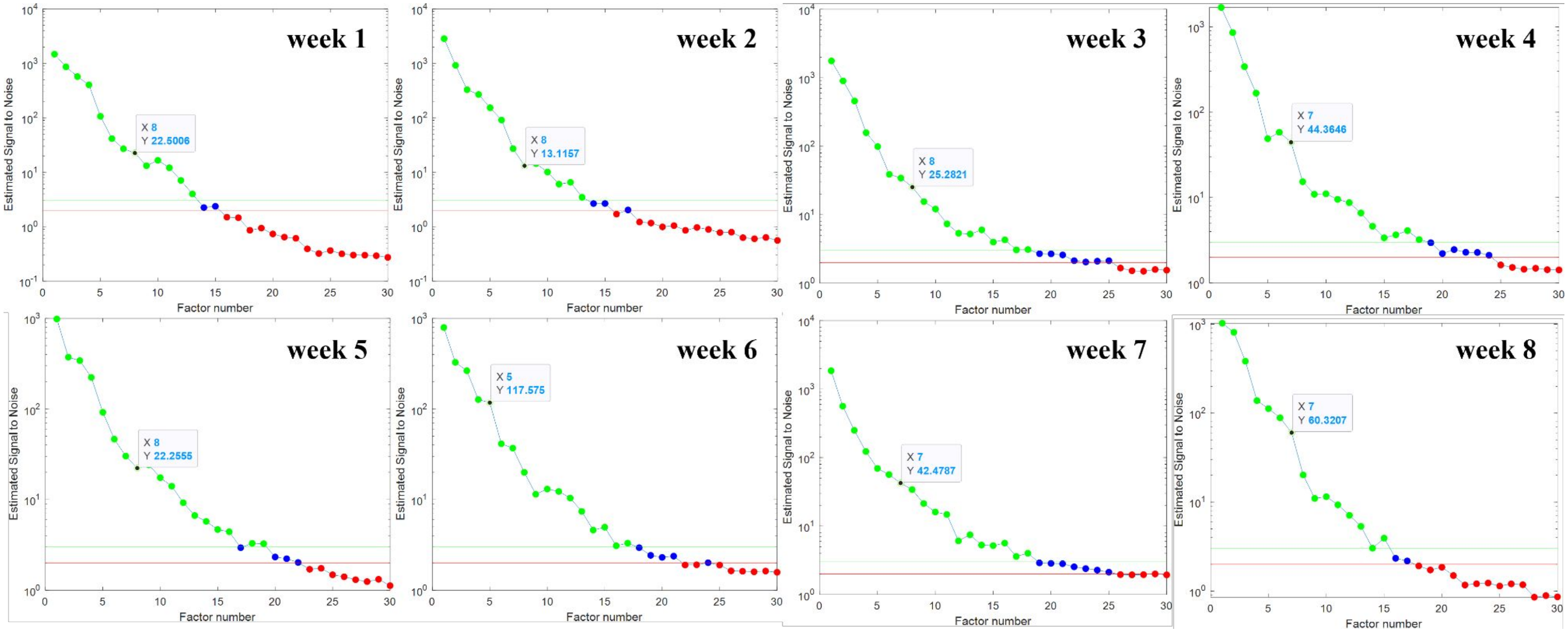

Figure S3. Estimated SNR for all PLS-DA models in Table 4.

Table S2. Permutation test results for all PLS-DA models in Table 4.

		**Random Prediction Probability (p)***	
**PLS-DA Model**	**Validation Type**	**Chlorine**	**Not Chlorine**
Week 1	Self-prediction	0.005	0.005
	Cross-validated	0.005	0.005
Week 2	Self-prediction	0.005	0.005
	Cross-validated	0.005	0.005
Week 3	Self-prediction	0.005	0.005
	Cross-validated	0.005	0.005
Week 4	Self-prediction	0.005	0.005
	Cross-validated	0.005	0.005
Week 5	Self-prediction	0.005	0.005
	Cross-validated	0.005	0.005
Week 6	Self-prediction	0.005	0.005
	Cross-validated	0.005	0.005
Week 7	Self-prediction	0.005	0.005
	Cross-validated	0.005	0.005
Week 8	Self-prediction	0.005	0.005
	Cross-validated	0.005	0.005

**If $p < 0.05$, the predictive classification of chlorine or not chlorine exposed is non-random.*

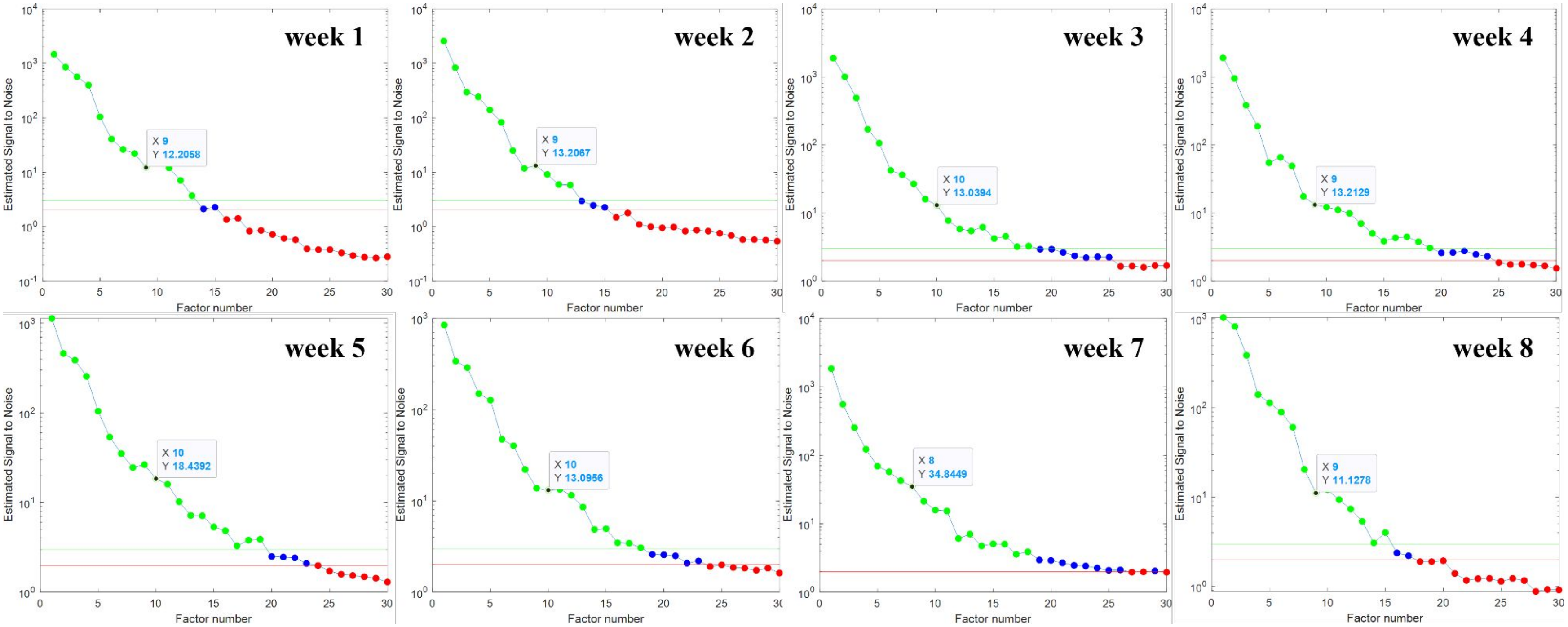

Figure S4. Estimated SNR for all PLS-DA models in Table 5.

Table S3. Permutation test results for all PLS-DA models in Table 5.

		Random Prediction Probability (p)*							
**PLS-DA Model**	**Validation Type**	**PBA in Chlorine**	**PBU in Chlorine**	**SBA in Chlorine**	**SBU in Chlorine**	**PBA in Other**	**PBU in Other**	**SBA in Other**	**SBU in Other**
Week 1	Self-prediction	0.005	0.005	0.005	0.005	0.005	0.005	0.005	0.005
	Cross-validated	0.005	0.005	0.005	0.005	0.005	0.005	0.005	0.005
Week 2	Self-prediction	0.005	0.005	0.005	0.005	0.005	0.005	0.005	0.005
	Cross-validated	0.005	0.005	0.005	0.005	0.005	0.005	0.005	0.005
Week 3	Self-prediction	0.005	0.005	0.005	0.005	0.005	0.005	0.005	0.005
	Cross-validated	0.005	0.005	0.005	0.005	0.005	0.005	0.005	0.005
Week 4	Self-prediction	0.005	0.005	0.005	0.005	0.005	0.005	0.005	0.005
	Cross-validated	0.005	0.005	0.005	0.005	0.005	0.005	0.005	0.005
Week 5	Self-prediction	0.005	0.005	0.005	0.005	0.005	0.005	0.005	0.005
	Cross-validated	0.005	0.005	0.005	0.005	0.005	0.005	0.005	0.005
Week 6	Self-prediction	0.005	0.005	0.005	0.005	0.005	0.005	0.005	0.005
	Cross-validated	0.005	0.005	0.005	0.005	0.005	0.005	0.005	0.005
Week 7	Self-prediction	0.005	0.005	0.005	0.005	0.005	0.005	0.005	0.005
	Cross-	0.005	0.005	0.005	0.005	0.005	0.005	0.005	0.005

	validated								
Week 8	Self-prediction	0.005	0.005	0.005	0.005	0.005	0.005	0.005	0.005
	Cross-validated	0.005	0.005	0.005	0.005	0.005	0.005	0.005	0.005

**If $p < 0.05$, the predictive classification of colorants that were chlorine or not chlorine exposed is non-random.*
